# Is the Cardiopulmonary Bypass Systemic Inflammatory Response Overestimated?

**DOI:** 10.21470/1678-9741-2018-0605

**Published:** 2018

**Authors:** Paulo Roberto B. Evora, Davi Freitas Tenório, Domingo M. Braile

**Affiliations:** 1 Editor-in-Chief Interim – BJCVS Faculdade de Medicina de Ribeirão Preto da Universidade de São Paulo (FMRP-USP), Ribeirão Preto, SP, Brazil.; 2 Resident in Cardiovascular Surgery at InCor - HCFMUSP Editorial Fellow BJCVS; 3 Editor-in-Chief – BJCVS Faculdade de Medicina de São José do Rio Preto (FAMERP), São José do Rio Preto, SP, Brazil and Universidade de Campinas (UNICAMP), Campinas, SP, Brazil.

## BJCVS Highlight

According to a survey by the University of Ottawa in 2009, we have surpassed the 50 million mark over the total number of scientific articles published since 1665, and approximately 2.5 million new scientific articles are published each year.

No doubt many of these articles will be ignored and others, depending on where they are published, will reach a wider audience. However, how can we be sure that the article produced with such care, which holds relevant scientific importance, will not be ignored? Moreover, which journals are able to reach your target audience?

One of the tools capable of predicting this and translating this subjective concept into a mathematical number is the impact factor^[1,2]^. The impact factor is the measure of how often a article article was cited in a given year. The more we read the article, the more likely it is to be quoted and the greater its impact factor.

Thus, last June the Journal Citation Reports (JCR) released the list of impact factors of scientific journals from around the world. Moreover, this year, the Brazilian Journal of Cardiovascular Surgery (BJCVS) has hit the world, reaching the impact factor of 0.805.

**Fig. 1:**
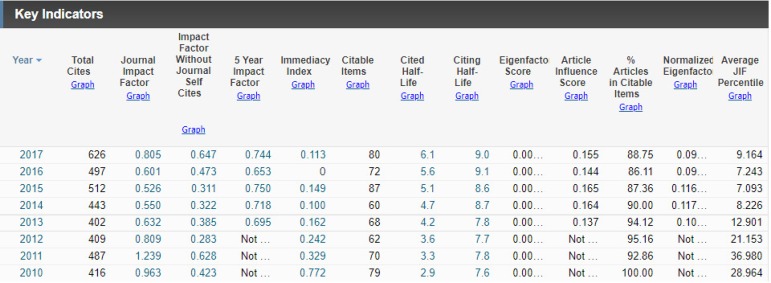
Latest impact factors of BJCVS.

This was the first year that the BJCVS, previously denominated Brazilian Journal of Cardiovascular Surgery, was cataloged with this denomination and, to the everyone's surprise, even with this critical obstacle, the BJCVS for the second consecutive year raised its index, and with an increase of more than 25% (Figure 1).

**Fig. 2:**
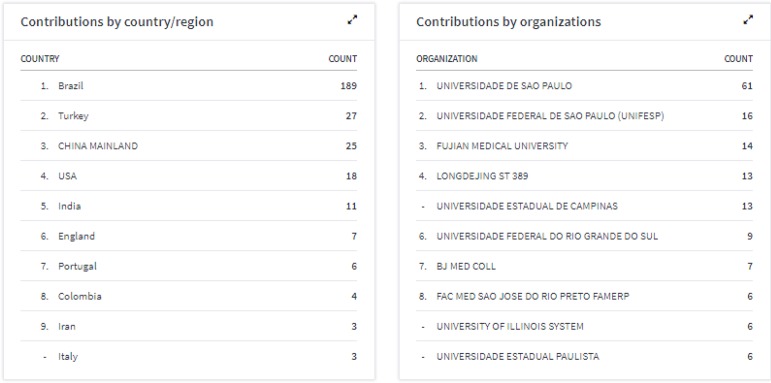
Division of BJCVS citations according to countries and institutions of origin.

The JCR also provides a division of citations according to the countries and institutions of origin of this citation; these data confirm the broad and international spectrum of readers that the BJCVS has attained (Figure 2).

This achievement reflects, above all, the dedication of a compromised editorial board that not only dedicates itself to selecting the best manuscripts, but also attempts to contribute to their improvement.

Good results like these only reinforce the journal's commitment to the dissemination of scientific production of merit and open doors to increasingly significant contributions.

## Is the Cardiopulmonary Bypass Systemic Inflammatory Response a Major Concern?

The first successful open-heart operation using cardiopulmonary bypass (CPB) was done by John Gibbon on May 6, 1953. The operation was the closure of an atrial septal defect. For a brief period (1955-1956), there were only two hospitals in the world where open heart surgery was being done on a daily basis: C. Walton Lillehei at the University of Minnesota and, 60 miles away, John Kirklin at the Mayo Clinic. From the systematized experience of these two institutions, began the era of modern open-heart surgery, followed by the evolution of the quality of CPB circuit components whose excellence, of course, was associated with considerable morbidity and mortality improvement. Then came the paradigm of the systemic inflammatory reaction triggered by the contact of the blood with the non-endothelial surface of the system, which is the main motivational aspect of this editorial.

The physiopathology of the CPB systemic inflammatory response is multifactorial, with no final consensus about its actual mechanism. It can be divided into two main phases: "early" and "late". The early phase occurs as a result of blood contact with non-endothelial surfaces ("contact activation"), and the late phase is driven by ischemia-reperfusion injury (I/R injury), endotoxemia, coagulation disorders, and reactions to heparin/protamine. The contact of blood with a non-physiological surface during CPB surgery is thought to induce a systemic inflammatory reaction syndrome (SIRS). CPB plastic components and the CPB procedures, per se, are thought to trigger the inflammatory response (early phase). This phase should occur in all patients undergoing cardiac surgery and, due to the disproportion of their severity with the number of surgeries performed worldwide, should not be the main cause of vasoplegic syndrome, characterized by systemic inflammatory reaction.

Today, this paradigm is strongly debated, mainly because an inflammatory response is still present in patients undergoing off-pump surgery. Also, O'Brien et al.^[3]^ reported that transcatheter aortic valve implantation (TAVI) (which reduces surgical trauma and avoids the need for CPB) does not attenuate the patients' innate inflammatory response. Many studies have shown that blood contact with the surgical wound may handle the inflammatory reaction (late phase). This hypothesis proposes that blood coming into contact with serous membranes (pleura and pericardium) causes fibrinolytic activity and increases bleeding, which agrees with recent advances in our knowledge and understanding about the association between coagulation and inflammation. As coagulation management during CPB is one of the most serious problems, it is possible that inadequate heparin use could handle inappropriate, imperceptible anticoagulation and therefore also trigger inflammation^[4,5]^. Thus, maintenance of pleural integrity in the dissection of internal thoracic arteries should be an interesting detail of the surgical technique.

Based on the literature overview and the worldwide cardiac surgery excellence, it seems possible that pathological inflammation during human CPB surgery is overestimated. However, when it occurs, it is associated with high morbidity and mortality, deserving constant vigilance. Despite being an unspecific marker of inflammation, C-reactive protein (CRP) and alkaline phosphatase are measured routinely by hospital laboratories and therefore would make useful markers in cardiac surgery. In addition, the relation neutrophil/lymphocyte (N/L) and the relation platelet/lymphocyte (P/L) have become useful inflammation biomarkers.

Finally, we would close the present editorial answering the provocative title question: Cardiopulmonary Bypass Systemic Inflammatory Response is Overestimated, Which is Better than Underestimated.

## Articles in this Issue

This issue of BJCVS presents a blind peer-reviewed selection of 16 papers that will surely please our readers. The vast majority related to various perioperative problems. We selected the articles by order of acceptance (11 original papers, 2 article reviews, and 2 elected case reports, 1 point of view).

Doctors Gabriel Romero Liguori and Luiz Felipe Pinho Moreira created this editorial series entitled "Operating with Data - Statistics for the Cardiovascular Surgeon". The series will merit five editorials, each one describing a different aspect of statistical analysis relevant for the cardiovascular surgeon, as follows: • Part I. Fundamentals of Biostatistics; • Part II. Association and Risk; • Part III. Comparing Groups; • Part IV. Correlations and Regression; • Part V. Survival Analysis. The BJCVS and its readers thank the commendable initiative of Drs. Liguori and Moreira. We clarify that, although with unusual characteristics of an opening editorial, we decided to consider the texts as Editorial to highlight them. The Part I was published on the 33.3 edition^[6]^.

**Paulo Roberto B. Evora** ^1^Editor-in-Chief Interim –
BJCVS Faculdade de Medicina de Ribeirão Preto da Universidade de
São Paulo (FMRP-USP), Ribeirão Preto, SP, Brazil.**Davi Freitas Tenório** ^2^Resident in Cardiovascular
Surgery at InCor – HCFMUSP Editorial Fellow BJCVS**Domingo M. Braile** ^3^Editor-in-Chief – BJCVS
Faculdade de Medicina de São José do Rio Preto (FAMERP),
São José do Rio Preto, SP, Brazil and Universidade de Campinas
(UNICAMP), Campinas, SP, Brazil.
